# The plague of our time:peer bullying and media portrayal

**DOI:** 10.1192/j.eurpsy.2026.10560

**Published:** 2026-07-31

**Authors:** D. Ocaklik Altunkiliç, S. Kökcealan, A. Okanlı

**Affiliations:** 1 Faculty of Health Sciences, Yeditepe University; 2 Istanbul Prof. Dr. Mazhar Osman Mental and Nervous Diseases Training and Research Hospital; 3 Faculty of Health Sciences, Istanbul Medeniyet University, Istanbul, Turks and Caicos Islands

## Abstract

**Introduction:**

Peer bullying is one of the most common and destructive social problems experienced during childhood and adolescence,and it is a serious public health issue that negatively affects individuals’ psychological,academic,and social development.Research indicates that approximately 20-30% of school-aged children experience some form of bullying.Individuals who experience bullying develop serious psychological issues such as depression, anxiety,social isolation,and suicidal thoughts;their academic performance declines,and the rate of early school dropout increases.The effects of bullying are not limited to the victims but can also have long-term consequences for the bullies and students who witness the incident.Furthermore,bullies are more likely to engage in criminal behaviour,antisocial behaviour,and substance use,and they tend to have lower occupational success in later life.In this context,peer bullying should be addressed not only as an individual issue but also as a social problem;it should be evaluated through a comprehensive approach by education systems,families,and policymakers.Media content that focuses on the psychological effects and psychiatric consequences of bullying plays an important role in raising social awareness.

**Objectives:**

This study was conducted qualitatively to explain the media’s portrayal of peer bullying in Turkey.

**Methods:**

Using the maximum diversity method,one of the purposive sampling methods,the relevant news from the top 10 most-read newspapers in Turkey from 2010,when peer bullying first came to the fore in Turkey,to the present day were examined.The first 8 newspapers were selected because they published news related to peer bullying,and a total of 53 news were examined and 9 main themes have been identified.

**Results:**

News content highlighted various factors that contribute to bullying, ranging from institutional shortcomings to socio-economic conditions, teacher attitudes to security gaps, and emphasised that constant exposure to bullying causes various negative emotions in individuals, such as withdrawal, feelings of worthlessness, guilt and anxiety,and in some cases, generates social reaction or awareness. Supportive factors such as families’ awareness levels, empathetic approaches, efforts to establish open communication, and school cooperation are key concepts in interventions against bullying. News content emphasises the need to raise awareness among teachers and parents in order to combat bullying. The content shows that bullying has not only psychological but also physical and behavioural consequences, and that high school is the level where bullying is most frequently experienced.

**Conclusions:**

The serious consequences experienced by individuals exposed to bullying include death,permanent physical damage and psychological trauma.Media coverage of topics such as preventing peer bullying,providing guidance,and support systems can have a more positive impact on society.

**Disclosure of Interest:**

D. Ocaklik Altunkiliç: None Declared, S. KÖKCEALAN Employee of: Contributed to the research process., A. Okanlı Consultant of: The researcher provided consultancy services for the study.

